# Assessment of serum amyloid A concentrations and biochemical profiles in lactating jennies and newborn Ragusano donkey foals around parturition and one month after foaling in Sicily

**DOI:** 10.1111/rda.14048

**Published:** 2021-11-29

**Authors:** Marilena Bazzano, Laura Bonfili, Anna Maria Eleuteri, Evelina Serri, Carmela Scollo, Yang Yaosen, Beniamino Tesei, Fulvio Laus

**Affiliations:** ^1^ School of Biosciences and Veterinary Medicine University of Camerino Matelica Italy; ^2^ Private Practitioner Catania Italy

**Keywords:** acute phase protein, donkey foals, jennies, serum amyloid A

## Abstract

A proper knowledge of biochemical parameters and inflammatory markers like serum amyloid A (SAA) is crucial in the monitoring of the first post‐partum period in equids. Since no information is available on SAA for donkeys at this stage, 50 animals including jennies (*n*.10) and newborn foals (*n*.10) within 48 hr from foaling, and jennies (*n*.10) and foals (*n*.20) after 30 days from parturition were enrolled in the study to assess routine biochemical profile including SAA. Jennies showed higher alkaline phosphatase and lower bilirubins and cholesterol at 30 days of lactation compared to post‐partum. Neonatal donkey foals showed significant higher concentrations of sodium, alkaline phosphatase, lactic dehydrogenase, blood urea nitrogen, creatinine and albumin within 48 hr of age, whilst higher values of phosphate and triglycerides were observed in older foals of 30 days of age. Significant higher SAA concentrations were recorded during the peripartum period in both jennies (25.95 ± 14.98 μg/ml) and newborn donkey foals (37.44 ± 19.75 μg/ml) compared to SAA values recorded in lactating jennies (2.38 ± 1.78 μg/ml) and in donkey foals (16.04 ± 18.14 μg/ml) at 30 days after parturition. The assessment of SAA in jennies and donkey foals around parturition and one month after foaling represents a valuable tool for the monitoring of health status during this stage when animals have to face with new challenges like the peak of lactation and extrauterine life adaptation respectively.

## 1 INTRODUCTION

Pregnancy, parturition and lactation represent specific conditions that significantly influence animal metabolism. The effects of peripartum period on haematological and biochemical parameters have been studied in mares (Bazzano et al., 2014; Mariella et al., 2014) and more recently also in jennies (Bonelli et al., 2016). Several biochemical parameters change in healthy pregnant subjects, thus confirming the need for proper reference ranges at this stage. Similarly, when assessing both equine and donkey foals, clinicians need to refer to specific reference ranges according to animal age (Sgorbini et al., 2013; Veronesi et al., 2014).

In the last years, the assessment of specific acute phase proteins has been added to routine haematological and biochemical analysis to support clinicians in the early recognition and differentiation of acute infection and inflammation from other more benign clinical disease (Kay et al., 2019). Serum amyloid A (SAA) is an acute phase protein of the apolipoprotein family (APP), mainly produced by the liver, that rapidly increases in response to inflammation (Long & Nolen‐Walston, 2020). Serum amyloid A is the only major positive APP in the horse as its concentrations are low or clinically undetectable in normal animals but rapidly increase from 10 up to 1,000 times following the onset of the acute phase response (Nolen‐Walston, 2015). Furthermore, SAA concentrations increase after 6 hr from the stimulus and decrease within 12 hr from the end of the disease because of its short half‐life (30–120 min) (Long & Nolen‐Walston, 2020). Several studies have investigated SAA concentrations in horses affected by respiratory disease, colic, orthopaedic conditions or undergoing surgical procedures (Witkowska‐Piłaszewicz, Żmigrodzka, et al., 2019). Recently, a certain interest has been directed at SAA modifications in mares affected by reproductive diseases (Coutinho da Silva et al., 2013). Studies have also been performed in healthy horses during different type of exercise (Piccione et al., 2016; Witkowska‐Piłaszewicz, Bąska, et al., 2019) or specific physiological state like peripartum in broodmares and neonatal foals (Chavatte et al., 1992; Krakowski et al., 2020; Stoneham et al., 2001). On the contrary, SAA concentrations have been scarcely investigated in domestic donkeys (Kay et al., 2019; McLean et al., 2016) and only one report exists on feral donkeys (Jerele et al., 2020).

Despite the increasing interest in donkey medicine and the need for specific reference range, no information is available approximately SAA values in healthy subjects. Considering the high potential of SAA for the early diagnosis of inflammatory diseases in equids, and the economic loss derived from a sick lactating jenny or a sick donkey foal, the early diagnosis of inflammatory disease in donkeys during this stage is of great importance.

Based on the current knowledge, the aim of this study was to investigate the main biochemical parameters and SAA levels in lactating Ragusana jennies and donkey foals reared for milk production during the first month after foaling.

## 2 MATERIALS AND METHODS

### 2.1 Animals

All animal housing, care and experimental procedures herein described were in accordance with the standards recommended by the EU Directive 2010/63/EU for experiments on animals. The research protocol was approved by Internal Animal Welfare Committee (approval number 5/2021).

A total of 50 Ragusana donkeys, reared in the same farm in Sicily, were enrolled in the study with the informed owner consent. All subjects underwent clinical examinations to confirm the health status, and only clinically healthy donkeys were included in the study. Animals were divided into four groups according to the time of sampling: Group A consisted of *n*.10 jennies within 48 hr from delivery, Group B included *n*.10 jennies at 30 days of lactation, Group C consisted of n.10 (6 males, 4 females) newborn donkey foals (within 48 hr from birth), and Group D included n.20 (11 males, 9 females) donkey foals (within 1 month of age) (Table 1). Jennies from Group A (mean age 7.5 ± 2 years, mean BCS (Evans & Crane, 2018) 2.8 ± 0.2) and Group B (mean age 7.7 ± 2.5 years, mean BCS 2.5 ± 0.2) were fed 6 kg/day dried grass hay and 0.5 kg/day concentrates. Donkey foals from both Group C and D were kept with their dams being allowed to nurse 24 hr/day during the experimental period. Animals were housed in individual straw‐bedded boxes during the first week after foaling, and then they were moved to common paddocks shared with other dams and foals. Furthermore, animals were allowed to pasture 8 hr/day and water was provided ad libitum.

**TABLE 1:** Mean values and standard deviations (*SD*) of age (days, d; years, y), body condition score (BCS, 5‐point scale) (Evans & Crane, 2018) or weight (kg), and sex (female, F; male, M) of Ragusano donkeys according to each group

	Age	BCS/weight	Sex
*Group A*	7.5 ± 2 years	2.8 ± 0.2 /5	F
*Group B*	7.7 ± 2.5 years	2.5 ± 0.2 /5	F
*Group C*	2 days	37 ± 2 kg	6 M, 4 F
*Group D*	30 days	55 ± 4 kg	11 M, 9 F

Note

Group A (*n*.10) jennies within 48 hr from delivery, Group B (*n*.10) jennies at 30 days of lactation, Group C newborn donkey foals (*n*.10) within 48 hr from birth, Group D (*n*.20) one‐month‐old donkey foals

Blood samples were collected during the routine veterinary procedures performed in the donkey farm, in the morning (9–10:00 a.m.) before feeding, during the month of August 2019. A single blood sample was drawn from the jugular vein of each animal into 10 ml tubes containing clot activators (VACUETTE; Greiner Bio‐One GmbH).

### 2.2 Laboratory analysis

After collection, blood samples were placed on ice and delivered to the laboratory within 2 hr. At the laboratory, samples were centrifuged for 10 min at 1,000 *g* (Universal 32, Hettich Zentrifugen, Germany) and the obtained sera were divided into two 1.5 ml aliquots and stored at −20°C until analysis.

Sera were tested for Potassium (K), Sodium (Na), Chloride (Cl), Calcium (Ca), Phosphorus (P), Calcium/Phosphorus ratio (Ca:P) blood urea nitrogen (BUN), γ‐glutamyl trasferase (GGT), glucose (Glu), creatinine (Cre), glutamic oxaloacetic transaminase (GOT), serum glutamic pyruvic transaminase (GPT), lactate dehydrogenase (LDH), Alkaline phosphatase (ALP), cholesterol (Chol), triglyceride (Trig), creatine kinase (CK), total bilirubin (tBil), direct bilirubin (dBil), indirect bilirubin (iBil), total protein (TP), albumin (Alb), globulins (G) Albumin/Globulin ratio (Alb:G), by using the automatic clinical chemistry analyser BT 3500 VET plus (Biotecnica Instruments, Rome, Italy).

Serum Amyloid A (SAA) has been detected in serum samples by a solid phase sandwich enzyme‐linked immunosorbent assay (ELISA) using the multispecies Tridelta PhaseTM range SAA kit (Cat. No. TP‐802, Tridelta Development Ltd.) (McDonald et al., 1991). Briefly, a monoclonal antibody specific for SAA was coated onto the wells of the microtiter strips provided. Samples at a dilution of 1:2000 and calibrators of known SAA content were incubated in micro‐wells at 37°C together with a horseradish peroxidase (HRP) labelled anti‐SAA antibody. Any SAA present was captured between the coated microplate and the labelled antibody. After washing steps, the chromogenic substrate 3,3′,5,5′‐tetramethylbenzidine was added. The resulting blue product is directly proportional to the amount of SAA present in the serum of donkeys. The reaction was stopped adding a stop solution and the intensity of colour was measured at 450 nm using a microtiter plate reader. The concentrations of the test samples derived from the calibration semi‐logarithmic standard curve and was expressed as mean concentration (µg/ml) ± *SE*. The intra (within) assay precision/reproducibility and the inter‐batch precision/reproducibility have been detected to validate the test, as indicated in the datasheet.

### 2.3 Statistical analysis

Data were analysed using statistical software Prism 8 (GraphPad Software Ltd).

Student's *t* tests were performed to highlight significant differences in studied blood parameters between Group A and B, and between Group C and D respectively. Values of *p* < .05 were considered statistically significant.

Statistical analysis was performed with one‐way analysis of variance (ANOVA), followed by the Bonferroni test using Sigma‐stat 3.1 software (SPSS, Chicago, IL, USA). Significantly different values (*p* < .05) were indicated in bold letters.

## 3 RESULTS

All jennies included in the study delivered at term (mean gestation length 355 ± 19 days), by spontaneous eutocic parturition, healthy viable foals.

Statistical analysis revealed significant higher concentrations of SAA values in jennies (Group A) and foals (Group C) at 48 hr from foaling compared with Group B of lactating jennies and Group D of one‐month‐old foals respectively. Studied biochemical parameters were found to differ between groups. ALP levels increased, whereas Chol, tBil, dBil, iBil decreased in Group B compared to Group A. Foals from group C showed higher serum concentration of Na, BUN, Crea, ALP, LDH, Alb and Ca/P ratio, and lower P and TG compared to Group D.

Mean values and standard deviations (*SD*) of biochemical parameters recorded for Group A and B are reported in Table 2 together with *p* values obtained from statistical analysis.

**TABLE 2:** Mean values ± standard deviations (*SD*) and statistical significances of biochemical parameters recorded for Ragusano jennies

Parameter	Units	Group A	Group B	*p* values
SAA	ug/ml	25.95 ± 2.39	14.99 ± 1.79	**<.001**
K	mEq/l	4.368 ± 0.36	4.648 ± 0.39	.056
NA	mEq/l	138.900 ± 4.18	139.6 ± 3.20	.340
CL	mEq/l	103.960 ± 2.92	104.04 ± 2.75	.475
CA	mg/dl	14.400 ± 0.74	14.94 ± 0.78	.065
P	mg/dl	3.659 ± 0.91	3.544 ± 0.46	.363
CA/P	ratio	4.216 ± 1.27	4.138 ± 0.67	.433
BUN	mg/dl	35.060 ± 5.01	36.13 ± 7.96	.362
CREA	mg/dl	1.498 ± 0.23	1.416 ± 0.20	.202
GOT	UI/l	17.763 ± 21.18	11.5112 ± 19.90	.252
GPT	UI/l	13.668 ± 2.86	18.473 ± 12.05	.118
GGT	UI/l	46.950 ± 17.75	52 ± 30.14	.327
ALP	IU/l	537.200 ± 72.51	661.8 ± 139.76	.**011**
CK	UI/l	406.400 ± 126.64	479.8 ± 157.70	.133
LDH	UI/l	319.600 ± 83.84	387.9 ± 249.30	.211
GLU	mg/dl	81.930 ± 25.66	68.07 ± 28.44	.134
CHOL	mg/dl	111.590 ± 22.39	95.03 ± 15.56	.**035**
TG	mg/dl	57.270 ± 26.56	53.46 ± 36.79	.397
DBIL	mg/dl	0.153 ± 0.05	0.0999 ± 0.01	.**002**
TBIL	mg/dl	0.286 ± 0.10	0.1699 ± 0.02	.**001**
IBIL	mg/dl	0.132 ± 0.05	0.0698 ± 0.01	.**001**
TP	g/dl	9.182 ± 0.66	9.102 ± 0.40	.373
ALB	g/dl	3.498 ± 0.21	3.306 ± 0.31	.063
GLOB	g/dl	5.686 ± 0.59	5.797 ± 0.34	.305

Note

Group A (*n*.10) jennies within 48 hr from delivery, Group B (*n*.10) jennies at 30 days of lactation. List of parameters: Serum Amyloid A (SAA), Potassium (K), Sodium (Na), Chloride (Cl), Calcium (Ca), Phosphorus (P), Calcium/Phosphorus ratio (Ca:P) blood urea nitrogen (BUN), γ‐glutamyl trasferase (GGT), glucose (Glu), creatinine (Cre), glutamic oxaloacetic transaminase (GOT), serum glutamic pyruvic transaminase (GPT), lactate dehydrogenase (LDH), Alkaline phosphatase (ALP), cholesterol (Chol), triglyceride (Trig), creatine kinase (CK), total bilirubin (tBil), direct bilirubin (dBil), indirect bilirubin (iBil), total protein (TP), albumin (Alb), globulins (G) Albumin/Globulin ratio (Alb:G). Significant (*p* < .05) *p* values were indicated in bold.

Mean values and *SD* of biochemical parameters together with *p* values recorded for group C and D are reported in Table 3.

**TABLE 3:** Mean values ± standard deviations (*SD*) and statistical significances of biochemical parameters recorded for Ragusano foals

Parameter	Units	Group C	Group D	*p* values
SAA	ug/ml	37.44 ± 19.76	16.04 ± 18.14	.**006**
K	mEq/l	3.967 ± 0.35	4.204 ± 0.57	.089
NA	mEq/l	128.7 ± 3.06	125.833 ± 3.54	.**018**
CL	mEq/l	95.37 ± 2.97	93.384 ± 2.92	.051
CA	mg/dl	12.34 ± 0.85	11.979 ± 0.76	.138
P	mg/dl	6.494 ± 1.14	7.252 ± 0.51	.**036**
CA/P	ratio	1.976 ± 0.50	1.659 ± 0.13	.**039**
BUN	mg/dl	26.62 ± 8.31	18.042 ± 5.32	.**006**
CREA	mg/dl	1.707 ± 0.36	1.262 ± 0.16	.**002**
GOT	UI/l	80.75 ± 58.75	80.288 ± 49.62	.492
GPT	UI/l	41.017 ± 57.46	14.284 ± 8.31	.088
GGT	UI/l	74.87 ± 21.71	66.811 ± 28.53	.202
ALP	IU/l	2,330.6 ± 1,062.84	867.474 ± 258.75	.**001**
CK	UI/l	416.6 ± 395.52	350.105 ± 170.69	.311
LDH	UI/l	457.3 ± 134.75	277.684 ± 130.64	.**001**
GLU	mg/dl	120.16 ± 22.40	111.868 ± 21.15	.174
CHOL	mg/dl	175.5 ± 35.62	176.526 ± 18.89	.467
TG	mg/dl	66.9 ± 11.82	85.189 ± 28.57	.**011**
DBIL	mg/dl	0.268 ± 0.14	0.331 ± 0.19	.163
TBIL	mg/dl	0.407 ± 0.17	0.531 ± 0.29	.079
IBIL	mg/dl	0.139 ± 0.09	0.202 ± 0.11	.058
TP	g/dl	5.82 ± 0.97	5.575 ± 0.50	.236
ALB	g/dl	3.163 ± 0.27	2.951 ± 0.26	.**030**
GLOB	g/dl	2.656 ± 0.88	2.623 ± 0.51	.458

Note

Group C newborn donkey foals (*n*.10) within 48 hr from birth, Group D (*n*.20) one‐month‐old donkey foals. List of parameters: Serum Amyloid A (SAA), Potassium (K), Sodium (Na), Chloride (Cl), Calcium (Ca), Phosphorus (P), Calcium/Phosphorus ratio (Ca:P) blood urea nitrogen (BUN), γ‐glutamyl trasferase (GGT), glucose (Glu), creatinine (Cre), glutamic oxaloacetic transaminase (GOT), serum glutamic pyruvic transaminase (GPT), lactate dehydrogenase (LDH), Alkaline phosphatase (ALP), cholesterol (Chol), triglyceride (Trig), creatine kinase (CK), total bilirubin (tBil), direct bilirubin (dBil), indirect bilirubin (iBil), total protein (TP), albumin (Alb), globulins (G) Albumin/Globulin ratio (Alb:G). Significant (*p* < .05) *p* values were indicated in bold.

## 4 DISCUSSION

Over the last years, several researchers investigated the haematological profile during the peripartum period in donkeys (Bonelli et al., 2016; Sgorbini et al., 2013; Veronesi et al., 2014); however, to the best authors knowledge, this is the first report including SAA assessment in the routine biochemical profile of lactating jennies and neonatal donkey foals.

Comparing our results with the existing literature, few parameters undergo significant modifications in jennies during lactation. The decrease in serum cholesterol at 30 days of lactation compared to the post‐partum observed in our study confirms previous findings in lactating jennies (Bonelli et al., 2016) and mares (Bazzano et al., 2014). According to Milonis and Polidori (Milonis & Polidori, 2021), despite donkey milk has lower fat percentage compared to other domestic species, the content of cholesterol in terms of g/100 g of fat ranges from 0.41 g/100 g to 0.97 g/100 g, that is even higher than cow and woman milk. Serum Alp values recorded in this study are higher compared to reference ranges for donkeys (Evans & Crane, 2018) and significantly increased at 30 days of lactation, in contrast with previous observations by Bonelli and colleagues (Bonelli et al., 2016). However, the tendency of serum Alp at increasing because of lactation has been demonstrated in other mammals like women and cows, by guessing that ALP originating from the mammary glands influenced serum Alp activity to some extent (Sato et al., 2005). Ragusana donkey is a breed mainly reared for milk production as jennies produce higher milk quantities compared to other breeds like the Amiata donkeys included in the study by Bonelli and colleagues (Bonelli et al., 2016). Mean values of bilirubins were found to be over the reference range in the imminent post‐partum and within the normal limits at 30 days of lactation. Despite we did not collect blood samples during late pregnancy, we can speculate that the higher values after parturition were due to cholestasis induced by the pregnant uterus as previously observed in periparturient mares (Bazzano et al., 2014; Mariella et al., 2014).

The biochemical profile of Ragusano donkey foals underwent significant modification over the first month of life. As already observed in Amiata and Martina Franca donkey foals, also in Ragusano donkey foals BUN and creatinine decreased during the first month of life. However, we recorded lower mean BUN concentrations compared to Veronesi et al. (2014) and Sgorbini and colleagues (Sgorbini et al., 2013). Similarly to Amiata and Martina Franca donkey foals, ALP concentration lowered during the first 30 days of life, despite we found higher mean concentrations compared to previous studies (Sgorbini et al., 2013; Veronesi et al., 2014). LDH tend to decrease significantly in Ragusano donkey foals whereas no significant modification was observed in Martina Franca ones (Veronesi et al., 2014). Serum TG were found to increase in older Ragusano foals, but no significant difference was found by other authors (Sgorbini et al., 2013; Veronesi et al., 2014). Amongst serum electrolytes, we observed a slight decrease in Na at 30 days of life compared to neonatal period, and a significant increase of *p* as already found in Martina Franca donkey foals (Veronesi et al., 2014). Differently from other authors who found no significant change in albumin serum concentrations, we observed a slight decrease in Ragusano donkey foals at 30 days post‐partum (Sgorbini et al., 2013; Veronesi et al., 2014).

Concerning SAA assessment in donkey species, we observed significant modifications both in jennies and donkey foals during the experimental period. Currently, the commonly accepted reference range for equids (0–20 ug/ml) has been assessed in healthy horses, with no sex‐related differences, but an age‐related effect has been reported with healthy neonatal foals (<1‐week‐old) showing mean SAA concentrations of 27.1 ug/ml within the 3rd day of life (Witkowska‐Piłaszewicz, Żmigrodzka, et al., 2019). In our study, clinically healthy foals within 48 hr of age had mean SAA of 37.44 ± 19.76 ug/ml. Similar SAA concentration have been obtained in previous studies on equine foals, by developing a sepsis score where SAA concentrations <30 ug/ml represented zero score, that is, no sepsis, whilst SAA >150 ug/ml could be given a high score for septic foals, values between 30 and 150 ug/ml intermediate and those <30 ug/ml zero score (Stoneham et al., 2001). In the present study clinically healthy donkey foals showed a higher SAA concentration compared to neonatal equine foals; this gap might be due to a physiological difference related to donkey species or the timing of sampling. According to our results, the peripartum period significantly affected SAA concentrations reaching mean values of 25.95 ± 2.39 ug/ml in jennies within the first 48 hr following parturition, and then decreased up to 14.99 ± 1.79 at 30 days of lactation. According to previous studies in pregnant mares, SAA concentration remained stable within the normal range during the 4 months before parturition but increased from 1 week before until 1 month after foaling, which may result from tissue damage during the displacement of the foetus. Significant increases in SAA concentrations were noted at 12 hr (0.7–305 mg/L) and 36 hr (0–1,615 mg/L) and returned to basal concentrations within 60 hr post‐partum. Other studies have shown no changes in SAA concentrations after parturition in mares carrying normal pregnancies and delivering normal foals (Witkowska‐Piłaszewicz, Żmigrodzka, et al., 2019).

Despite the difference in methods can influence SAA assessment amongst studies, SAA concentrations tend to increase following parturition in mammals as this APP is involved in the process of parturition. According to a recent study by Gan and colleagues (Gan et al., 2020), SAA released by the placenta may participate in the onset of labour irrespective of infection by stimulating the expression of parturition‐pertinent inflammatory factors with consequently increased production of proinflammatory cytokines and PGF2α in the placenta. According to our results, healthy foals showed slightly higher SAA concentrations compared to jennies within 48 hr from foaling that, in the authors’ opinion, could be due to an early adaptation phase of new‐borns to extrauterine life.

The present study presents some limitations: sample size was consistent for run statistical evaluations but not large enough to consider the obtained data suitable for reference range, and the animals included in the study belonged to a single donkey farm. Further studies with larger sample size are necessary in order to define a threshold for health and disease.

## 5 CONCLUSIONS

The assessment of SAA in clinically healthy jennies and donkey foals approximately parturition and the peak of lactation provides useful information approximately the monitoring of health status in these equids reared for milk production (Bordonaro et al., 2013). Early detection of neonatal and post‐partum diseases is to present a diagnostic challenge especially in donkey species. Because of their stoicism, clinical signs in these equids are often subtle and nonspecific and general conditions can worse rapidly. More robust reference ranges for SAA are needed to be used in clinical practice; despite this, the information herein provided could represent an initial help in early diagnosis and prompt therapeutical intervention that may have a major influence on jenny and foal survival, as well as on milk production, with significant economic consequences for donkey farmers.

## CONFLICT OF INTEREST

Authors declare no conflict of interest.

## Data Availability

Research data are not shared.
